# Multi-Scale Topology Optimization of Femoral Stem Structure Subject to Stress Shielding Reduce

**DOI:** 10.3390/ma16083151

**Published:** 2023-04-17

**Authors:** Zhongmin Xiao, Longfei Wu, Wenqiang Wu, Ruizhi Tang, Jietao Dai, Dachang Zhu

**Affiliations:** School of Mechanical and Electrical Engineering, Guangzhou University, Guangzhou 510006, China

**Keywords:** topology optimization, multi-scale conditions, femoral stem, stress shielding, finite element analysis

## Abstract

Hip replacement femoral implants are made of substantial materials that all have stiffness considerably higher than that of bone, which can cause significant bone resorption secondary to stress shielding and lead to severe complications. The topology optimization design method based on the uniform distribution of material micro-structure density can form a continuous mechanical transmission route, which can better solve the problem of reducing the stress shielding effect. A multi-scale parallel topology optimization method is proposed in this paper and a topological structure of type B femoral stem is derived. Using the traditional topology optimization method (Solid Isotropic Material with Penalization, SIMP), a topological structure of type A femoral stem is also derived. The sensitivity of the two kinds of femoral stems to the change of load direction is compared with the variation amplitude of the structural flexibility of the femoral stem. Furthermore, the finite element method is used to analyze the stress of type A and type B femoral stem under multiple conditions. Simulation and experimental results show that the average stress of type A and type B femoral stem on the femur are 14.80 MPa, 23.55 MPa, 16.94 MPa and 10.89 MPa, 20.92 MPa, 16.50 MPa, respectively. For type B femoral stem, the average error of strain is −1682με and the average relative error is 20.3% at the test points on the medial side and the mean error of strain is 1281με and the mean relative error is 19.5% at the test points on the outside.

## 1. Introduction

When the function of the femoral stem is damaged or lost due to hip joint disease or accident, it will bring great pain and inconvenience to people’s work and life. In cases of osteoarthritis and trauma injuries involving the hip joint that can impact the quality of life of individuals, total hip arthroplasty is one of the main surgical procedures [1]. Due to the difference between the material stiffness of artificial femur (such as alumina ceramic, titanium alloy, zirconium niobium alloy and carbon fibre composite materials) and the bone stiffness, the stress shielding phenomenon will lead to the femur loosening, falling off and even leading to fracture and other serious consequences [2,3,4]. A change in bone mechanics transmission route due to femur bone damage causes the stress shielding effect. The formation of a porous structure provides a better solution to the problem of stress shielding effect reduction [5]. A femoral stem design featuring a diamond cubic lattice with a porous structure was proposed in [6]. Jette et al. [7] produced two femoral stems via laser powder-bed fusion using Ti-6Al-4V alloy with a fully dense and diamond cubic lattice structure in its core. The comparison of the force-displacement diagram and displacement and strain field tests showed that the femur with porous prosthesis had less stress absorption than that with a dense counterpart. The femur stem of the homogenous porous and functionally graded porous stems incorporating a body-centered cubic structure can be used as an alternative to the dense stems. Its stress is 12∼34% lower than that of dense stems and the highest micromotions (105–147) μm were observed for stems of 80% overall porosity [8]. Homogeneous and functionally graded porous structures were introduced into titanium alloy femoral stems in [9]. It is worth noting that physiological loads were selected in the stress transfer experiment. Repetitive loading is one factor that leads to fatigue failure of the implanted stems. The porosities determined under repeated loading are the femoral stem structure closest to the intact bone [10]. Porous femoral stems with four different grading orientations along the axial and radial directions of the femoral stem were evaluated in [11]. The finite element (FE) models of the TKA knee with four different structures in the middle segment of the tibial stem (i.e., solid, cubic, truncated cubic and octahedral structures) were constructed and the Von Mises stress was analyzed [12]. It was found that a porosity of 47.3% of the body-centered cubic structure exhibits the closest stiffness (469 N/mm) to an intact bone (422 N/mm) [13]. Studies have shown that porous femur implants with gradient Poisson’s ratio distribution have smoother stress–strain distribution, effectively solving the mechanical mismatch problem [14,15]. The safety and effectiveness of porous femoral stems depend not only on the characteristic of the porous structure but also on the macro design of the femoral stem, such as the distribution of the porous structure, the stem geometric shape, the material and the manufacturing process [16,17,18,19].

Providing a femoral stem structure that mimics the mechanical properties of human bone is ideal for total hip replacement [20] and joint prosthesis survival is associated with the quality of surrounding bone [21]. MessinaFinite element analysis (FEA) is a computerized method that analyzes the effect of forces applied to a structure with a complex shape. By using computed tomography (CT), three-dimensional (3D) models of Dorr Type A femur and five commonly used primary total hip arthroplasty cementless stem designs were developed in [22] and the femoral strain along the implant bone was analyzed with FEA. In addition, the selection of materials [23,24,25], the geometry of the femur stem [26,27,28,29] and the analysis and calculation of stress [30,31] have been proposed. However, maximizing the mechanical properties of humans is a technical problem faced by total hip replacement surgery. The solid isotropic material with penalization (SIMP) method was adapted in [32] and two hip stems were designed for more natural load transfer and reducing strain shielding. Considering that the stress shielding effect is caused by the change in the mechanical transfer path caused by the femur injury, the density distribution of the material should be established according to the different loads of the material at each location. Therefore, based on the structure constructed by the individualized parameters and the CT data of the human femur, a multi-scale model of the femur stem based on the homogenous topology optimization method was proposed [33]. To achieve a balance between low stiffness and strength and reduce the stress shielding caused by high stiffness, a numerical optimization design method of the grid-filled rod was proposed in [34]. Using an optimization methodology that combined an octet-truss porous structure with density-based topology optimization, a compound sleeve and stem prosthesis were designed to improve the stability of traditional prosthetics [35]. Because of the above analysis, a novel topology optimization method is proposed in this paper. The main contributions of this paper are summarized as follows:

1. Considering the mechanical characteristics and structural composition of the femoral stem and the flexibility variation amplitude of the femoral stem structure, multi-scale parallel topology optimization is proposed;
2. Compared with the traditional topology optimization method (SIMP), the average stress of the type B femoral stem is 16.10 MPa and the stress shielding reduction is 20.3%;

The rest of the paper is structured as follows: In Section 2, the topology optimization model of the femoral stem is built. Two types of femoral stem with topological structure are derived in Section 3. The corresponding simulations and experiments are given in Section 4 and Section 5. Finally, conclusions are drawn in Section 6.

## 2. Method Description and Topological Model

Considering the correspondence between the density of femoral stem units, the stress transfer can be expressed in the form of a density function of continuous variables, satisfied with

(1) $$E_{i}=E_{\min}+l_{i}^{p}\left(E_{0}-E_{\min}\right)$$

where $E_{i}$ is the elastic modulus of the *i*th element after interpolation, $E_{0}$ is the elastic modulus of solid part material, $E_{min}$ is the elastic modulus of porous part material, $l_{i}$ is the relative density of the *i*th element and *p* is the penalty factor. With minimum compliance as the optimization objective, element density in the optimized interval as the design variable and volume fraction as the constraint condition, the mathematical model of SIMP interpolation is given by

(2) $$\left\{\begin{array}{c} \min C(L)=FU\\ \;s.t.\;KU=F\\ \frac{V}{V_{0}}-f_{0}=\sum_{i}^{N}\left(l_{i}v_{i}\right)/V_{0}-f_{0}\leq 0\\ 0<l_{\min}\leq l_{i}\leq 1 \end{array}\right.$$

where *C* is the structural compliance, *L* is the element relative density, *F* is the load vector, *U* is the displacement vector, *K* is the global stiffness matrix, $V_{0}$ is the initial structural volume, *V* is the optimized structural volume, $v_{i}$ is the element volume, $f_{0}$ is the volume fraction, $l_{min}$ is the design variable minimization and $l_{min}=0.001$, *N* is the total number of discrete units in the design domain.

The microscopic composition of the topological configuration determines the macroscopic mechanical properties, while the macroscopic mechanical properties determine the structural composition of the microstructure. The multi-scale parallel topology optimization comprehensively considers the relationship between the above two, and yields

(3) $$\left\{\begin{array}{c} \;(\mathrm{Find}:\;\rho_{M}^{i},\rho_{m}^{j}\left(i=1,2,\ldots ,N_{M};j=1,2,\ldots ,N_{m}\right)\\ \;\mathrm{Min}:\;c=\frac{1}{2}\int_{\Omega_{M}}D_{M}\left(\rho_{M}^{i},\rho_{m}^{j}\right)\varepsilon \left(u_{M}\right)\varepsilon \left(u_{m}\right)d\Omega_{M}\\ \;S.t.\;\alpha \left(u_{v},v_{v},D_{M}\right)=l\left(v_{M}\right)\\ \alpha \left(u_{m},v_{m},D_{m}\right)=l\left(v_{m}\right)\\ G_{M}\left(\rho_{M}\right)=\int_{\Omega_{M}}v_{0}\rho_{M}d\Omega_{M}-V_{M}\leq 0\\ G_{m}\left(\rho_{m}\right)=\int_{\Omega_{m}}v_{0}\rho_{m}d\Omega_{m}-V_{m}\leq 0\\ 0<\rho_{M}^{\min}\leq \rho_{M}^{i}\leq 1\\ 0<\rho_{m}^{\min}\leq \rho_{m}^{j}\leq 1 \end{array}\right.$$

where *c* is the objective function (average structural compliance), $\rho_{M}$ and $\rho_{m}$ are design variables at macro and micro scales, $N_{M}$ and $N_{m}$ are the numbers of macro-structure elements and micro-structure elements, respectively, $\Omega_{M}$ is the design domain of macro-structure and $\Omega_{m}$ is the design domain of micro-structure, $G_{M}$ and $G_{m}$ are the macro- and microscopic volume constraint, respectively, $V_{M}$ is the maximum volume fraction of the microscopic structure of the material, $u_{M}$ is the macroscopic stress domain, $v_{M}$ is the virtual displacement domain of macroscopic structure, $u_{m}$ is the microscopic displacement domain, $v_{m}$ is the virtual displacement domain of the microscopic structure, α is the double linear energy function and *l* is the linear load function. On the macroscopic scale, the equilibrium equation of state is given by the principle of virtual work, satisfied with

(4) $$\alpha \left(u_{M},v_{M},D_{M}\right)=\int_{\Omega_{M}}D_{M}\left(\rho_{M},\rho_{m}\right)\varepsilon \left(u_{M}\right)\varepsilon \left(v_{M}\right)d\Omega_{M}$$

(5) $$l\left(v_{M}\right)=\int_{\Omega_{M}}fv_{M}d\Omega_{M}+\int_{\tau_{M}}hv_{M}d\tau_{M}$$

(6) $$\alpha \left(u_{m},v_{m},D_{m}\right)=\int_{\Omega_{m}}D_{m}\left(\rho_{m}\right)\varepsilon \left(u_{M}\right)\varepsilon \left(v_{m}\right)d\Omega_{m}$$

(7) $$l\left(v_{m}\right)=\int_{\Omega_{m}}D_{m}\left(\rho_{m}\right)\varepsilon \left(u_{m}^{0}\right)\varepsilon \left(v_{m}\right)d\Omega_{m}$$

where *f* is the physical strength in the macro-structure, *h* is the boundary traction force on the second boundary $\tau_{M}$ of the macro-structure, $D_{M}$ and $D_{m}$ are stiffness tensors of the macro-structure and micro-structure, respectively. By the definition of (1), we obtain

(8) $$D_{M}=\left[E_{\min}+\rho_{M}^{p}\left(E_{0}-E_{\min}\right)\right]D^{H}$$

(9) $$D_{m}=\left[E_{\min}+\rho_{m}^{p}\left(E_{0}-E_{\min}\right)\right]D^{0}$$

where $D^{0}$ is a constitutive tensor of material, and $D^{H}$ is the homogenizing stiffness tensor and is given by

(10) $$D^{H}=\frac{1}{\left|\Omega_{m}\right|}\int_{\Omega_{m}}D_{m}\left(\rho_{m}\right){\left(\varepsilon \left(u_{m}^{0}\right)-\varepsilon \left(u_{m}\right)\right)}^{2}d\Omega_{m}$$

The sensitivity of the macro-scale objective function and macroscopic volume constraints to the design variables can be calculated as follows.

(11) $$\frac{\partial C}{\partial \rho_{M}}=-\frac{1}{2}\int_{\Omega_{M}}p{\left(\rho_{M}\right)}^{p-1}D^{H}\left(\rho_{m}\right)\varepsilon \left(u_{M}\right)\varepsilon \left(u_{m}\right)d\Omega_{M}$$

(12) $$\frac{\partial G_{M}}{\partial \rho_{M}}=\int_{\Omega_{M}}v_{0}d\Omega_{M}$$

The sensitivity of the micro-scale objective function and micro-volume constraints to the design variables can be calculated as follows.

(13) $$\frac{\partial C}{\partial \rho_{m}}=-\frac{1}{2}\int_{\Omega_{M}}\rho_{M}^{p}\frac{\partial D^{H}\left(\rho_{m}\right)}{\partial \rho_{m}}\varepsilon \left(u_{M}\right)\varepsilon \left(u_{m}\right)d\Omega_{M}$$

(14) $$\frac{\partial G_{m}}{\partial \rho_{m}}=\int_{\Omega_{m}}v_{0}d\Omega_{m}$$

Differentiating (10) to $\rho_{m}$, we obtain

(15) $$D^{H}=\frac{1}{\left|\Omega_{m}\right|}\int_{\Omega_{n}}D_{m}\left(\rho_{m}\right){\left(\Delta \varepsilon \left(u_{m}\right)\right)}^{2}d\Omega_{m}$$

where $\Delta \varepsilon \left(u_{m}\right)=\varepsilon \left(u_{m}^{0}\right)-\varepsilon \left(u_{m}\right)$.

## 3. Design of Prosthetic Femoral Stem

**Figure 1:**
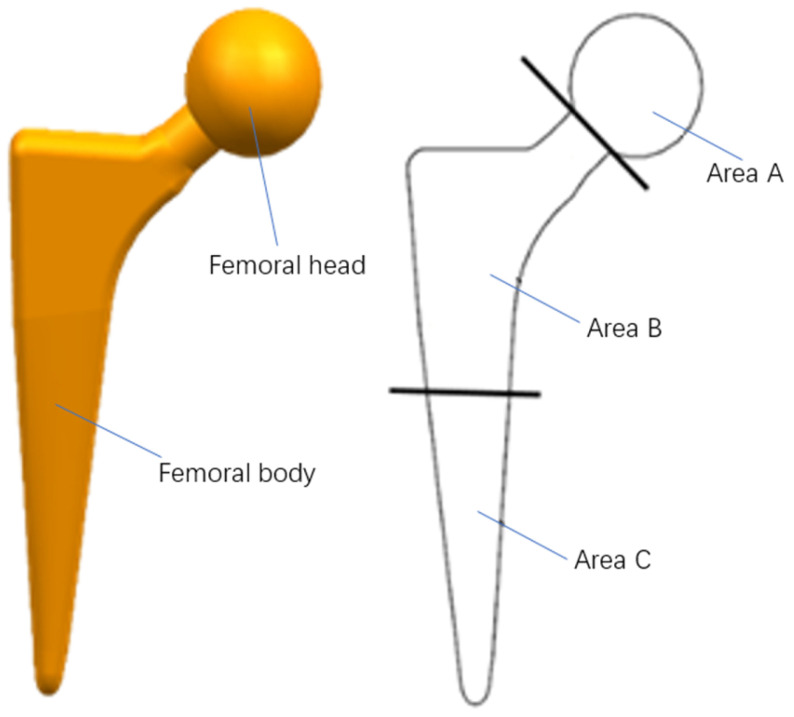
Structure of femoral stem.

The prosthetic femoral stem comprises two parts: the femoral head and the femoral body. The femoral head material is a mostly non-metallic composite material with a hard surface and scratch resistance and its shape is primarily spherical or oval. The neck of the femur has a smooth, solid surface and is usually integrated with the main shaft of the femur. We focus on the structural design of the Area B femoral stem. The structure of the femoral stem is shown in Figure 1.

**Figure 2:**
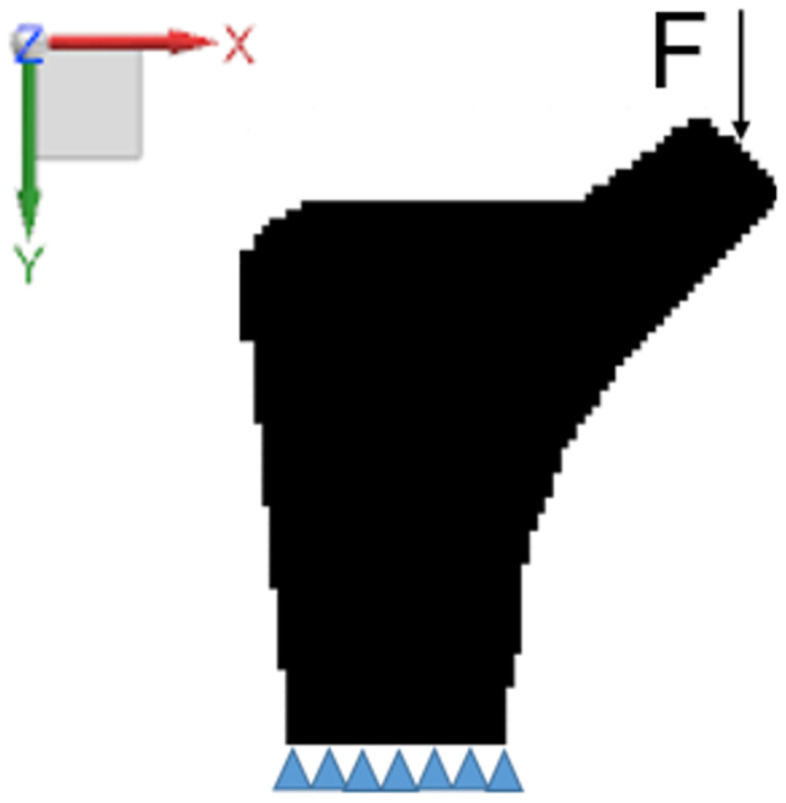
Design domain and boundary conditions.

The *B* region of the femur stem is taken as the design domain and the four-node element discrete structure is adopted in the two-dimensional space. The discrete structure of the design domain is 2928 elements, the element side length is set to 1, the material’s Poisson’s ratio is μ=0.3, the material’s elastic modulus is E=1, the penalty parameter is p=3, the filter radius $r_{min}=1.5$ and the *X* and *Y* directions of the bottom node are constrained. A concentrated load along with the *Y* axis is applied at the midpoint of the femoral neck end face, as shown in Figure 2.

**Figure 3:**
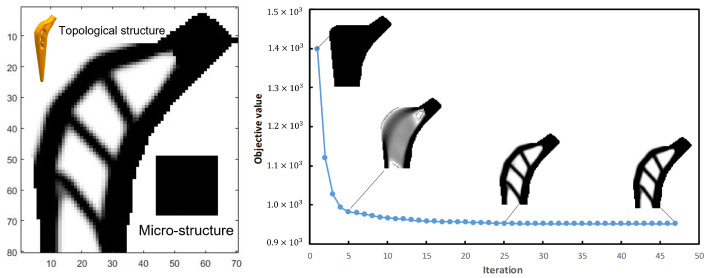
Topological configuration, micro-structure and solid model of A-type femoral stem.

The femur stem’s structure topology is carried out using SIMP and parallel optimization combined with the above initial parameters and boundary conditions. The volume fraction $f_{0}=0.6$ in the design domain of the SIMP method, the volume fraction $G_{M}=0.6$ in the macro-design domain and the volume fraction $G_{m}=0.6$ in the micro-design domain of parallel topological method are assumed to be a single type porous structure with periodic distribution in the macro-design domain. Using the SIMP method, the objective function is 951.7 after 57 iterations and we called it the A-type femoral stem. Its topological configuration, micro-structure and solid model are shown in Figure 3.

**Figure 4:**
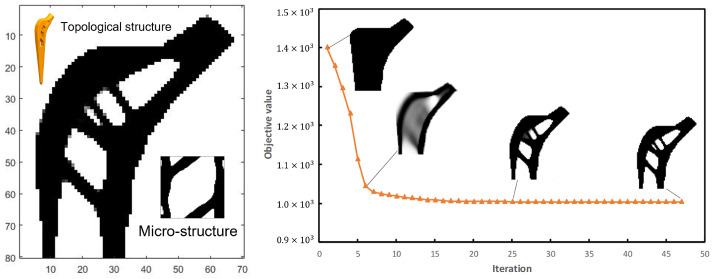
Topological configuration, micro-structure and solid model of B-type femoral stem.

The objective function is 1004.1 after 87 iterations with the parallel optimization method and we called it the B-type femoral stem. Its topological configuration, micro-structure and solid model are shown in Figure 4.

## 4. Simulations

### 4.1. Effect of Load Direction Change on Structure

In everyday life, the direction of the load on the femur changes frequently. Therefore, it is necessary to consider the influence of the direction change of load force on structural flexibility and overall stress. The proximal femur is affected by simultaneous loading force and muscle force, which is the main force.

**Figure 5:**
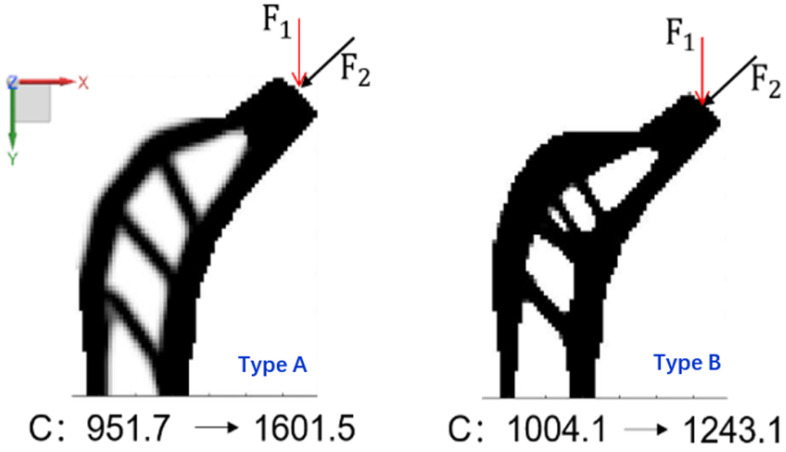
Variation of structural compliance when load force direction changes.

Step 1. Change load force direction. For the type A and type B femoral stems, the load force changes from $F_{1}$, the direction along the *Y* axis, to $F_{2}$, the direction at a 45-degree angle to the *Y* axis, as shown in Figure 5.

The compliance of the type A femoral stem structure changed from 951.7 to 1601.5 and the compliance of the type B femoral stem structure changed from 1004.1 to 1243. Although the initial compliance of the type A femoral stem structure is smaller than that of the type B femoral stem structure, when the load force direction changes, the compliance change of the latter is smaller than that of the former; this indicates that the sensitivity of type B femoral stem structure to load force direction change is lower than that of type A femoral stem structure to load force direction change.

**Figure 6:**
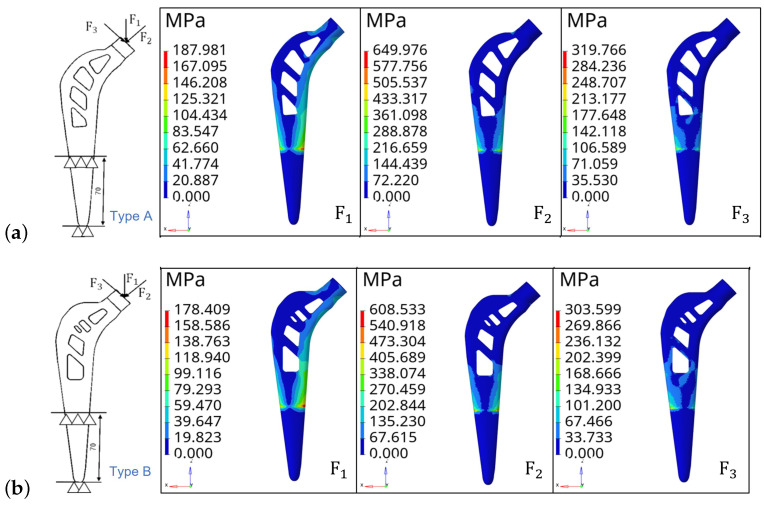
Constraints, vector load force and stress nephogram. (**a**) Type A. (**b**) Type B.

Step 2. Stress nephogram analysis. The part of the femoral stem between the lower end and the 70 mm section is set with fixed constraints. Two femoral stem structures are analyzed under three vector load forces: the size of $F_{1}$ is 2300 N, the direction along the *Y* axis, the size of $F_{2}$ is 2300 N and the direction of $F_{2}$ is the 45-degree angle from the *Y* axis, the size of $F_{3}$ is 2300 N and the direction of $F_{3}$ is −45 degree angle from the *Y* axis. The stress nephogram is shown in Figure 6.

Figure 6 shows that for type A femoral stem, the maximum stresses are 187.98 MPa, 649.97 MPa and 319.76 MPa when the load forces are $F_{1}$, $F_{2}$ and $F_{3}$, respectively. The maximum stresses for type B femoral stem are 178.40 MPa, 608.53 MPa and 303.59 MPa when the load forces are $F_{1}$, $F_{2}$ and $F_{3}$, respectively. It can be seen that the maximum stress generated by the type B femoral stem is smaller than that of the type A femoral stem when the load direction changes. Therefore, it is further indicated that the type B femoral stem has better stability and reliability against the change of load force direction.

### 4.2. Stress Analysis with Multi-Condition

The actual load on the femoral stem in daily human life is complicated. In general, the proximal end of the femur is subject to hip joint contact force, abductor muscle force, proximal fascia lata force, distal fascia force and muscle force. During the force analysis, the main forces were the hip joint contact and abductor muscle force, which significantly influenced the femur. At the same time, the three typical behaviors of standing on one leg and abduction at the proximal end of the femur are taken as working conditions. The load size and direction generated by the three working conditions are shown in Table 1.

**Figure 7:**
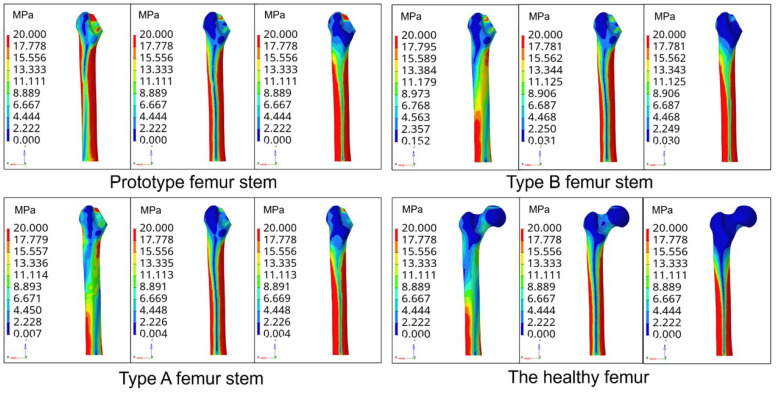
Stress nephogram of the femur stem under three conditions.

Not taking into account the slight displacement of the femur position after the hip replacement surgery, the femur and the prosthesis femur stem are subject to three conditions of stress: condition 1 is the standing condition on one leg, condition 2 is the abductive and condition 3 is addictive. The material density of the femur is set as $1.2\;\mathrm{Kg}/m^{3}$, the elastic modulus as 16.9 GPa and Poisson’s ratio as 0.26. The stem material of the femur is composite carbon fiber, with an elastic modulus of 23 GPa and Poisson’s ratio of 0.3. Through the finite element analysis, the equivalent stress nephogram of the prototype femoral stem, type A femoral stem and type B femoral stem on the femur and normal femur are obtained under three conditions, as shown in Figure 7.

**Figure 8:**
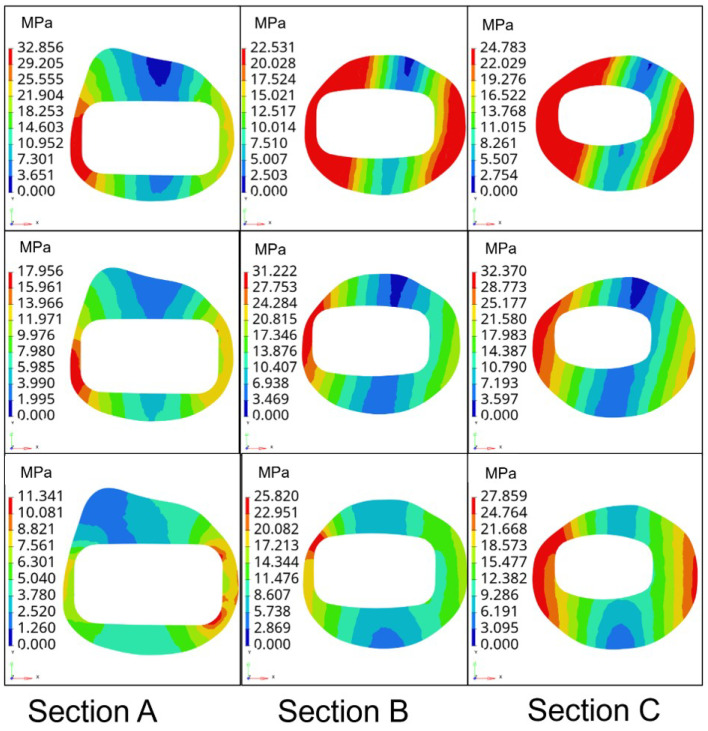
Stress nephogram distribution of prototype femur stem cross section.

Figure 8 shows that the femur corresponding to the prototype femur has a small amount of stress concentration at the proximal end of the femur. In contrast, the stress distribution of the type A and type B femur stems is more uniform, with less stress concentration. In the one-leg standing state, the high-stress zone of a healthy femur is located in the left region of the bottom of the femur, the middle-stress zone is located in the middle part of the femur and the low-stress zone is located in the proximal end of the femur. In contrast, the high-stress zone of the femur equipped with the prototype femur is mainly distributed on both sides of the femur. The stress distribution of the femur equipped with the type A and type B femur stems showed a gradual decrease from the distal end to the proximal end. The stress distribution is more similar to that of a healthy femur. Under both abduction and adduction conditions, the proximal stress of the femur equipped with the prototype femur is higher than that of the femur equipped with types A and B. In contrast, the stress distribution of the femur equipped with the type A and type B femurs is similar.

**Figure 9:**
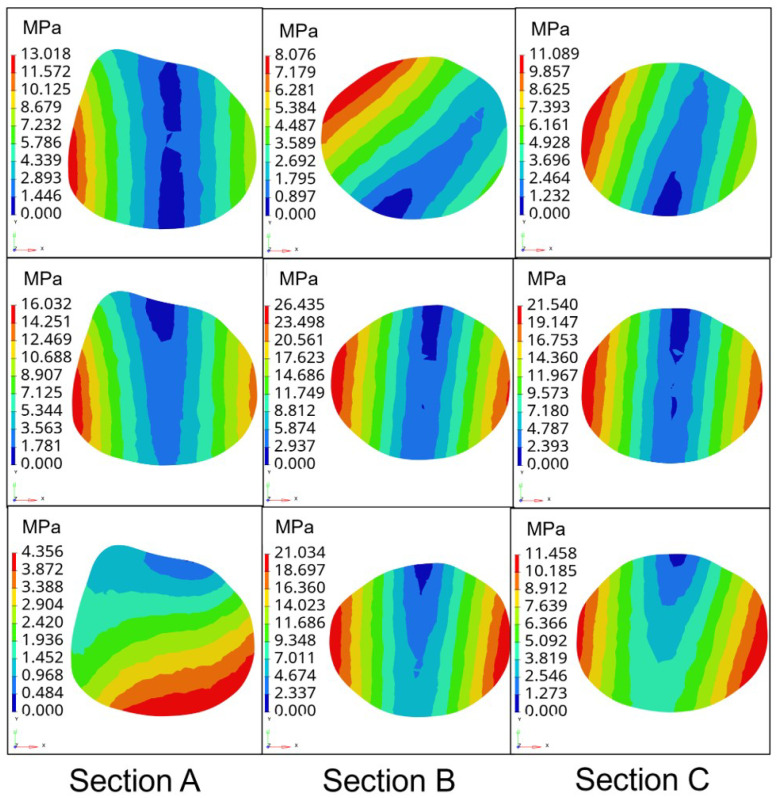
Stress nephogram distribution of three types of femur stem with three conditions.

Further, to verify the better biocompatibility of type B femoral stem compared with the femoral stem of the prototype, type A and healthy, the stress magnitude and distribution of three types of femoral stem on three sections of femur under three conditions are proposed under the same boundary conditions, as shown in Figure 9.

**Figure 10:**
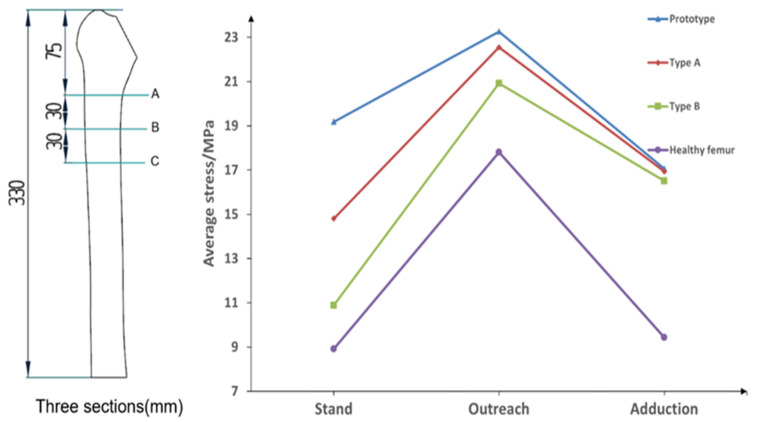
Average stress on the femur under different types of femoral stem under three conditions.

The division of three sections of the femur and the average stress values of sections under three conditions are shown in Table 2 and Figure 10.

Figure 10 shows that in the state of standing on one leg, the average stress on the femur of the prototype femur and the type A femur stem are 19.18 MPa and 14.81 MPa, respectively, while the average stress on the femur of type B femur stem is 10.89 MPa, which is closest to the average stress of the healthy femur in the second state. Under the extended condition, the average stress on the prototype femur stem and type A femur stem are 23.26 MPa and 22.55 MPa, respectively, and the average stress on the femur of the type B femur stem is the lowest among the three. In the state of adduction, the average stress on the prototype and type A femur is similar to 17.06 MPa and 16.94 MPa, respectively, while the average stress on the femur of type B femur stem is 16.50 MPa, showing little difference among the three as a whole. Compared with the other two types of the femoral stem, the type B femoral stem can better prevent the femur from generating internal stress and reduce the probability of postoperative fracture. Moreover, the average stress value of the type B femur stem on the femur is the closest to that of a healthy femur under three conditions, indicating that its mechanical properties are similar to those of a healthy femur, and has better biocompatibility and is more conducive to postoperative rehabilitation and curing of patients.

## 5. Experiments

### 5.1. Experimental Process and Results

**Figure 11:**
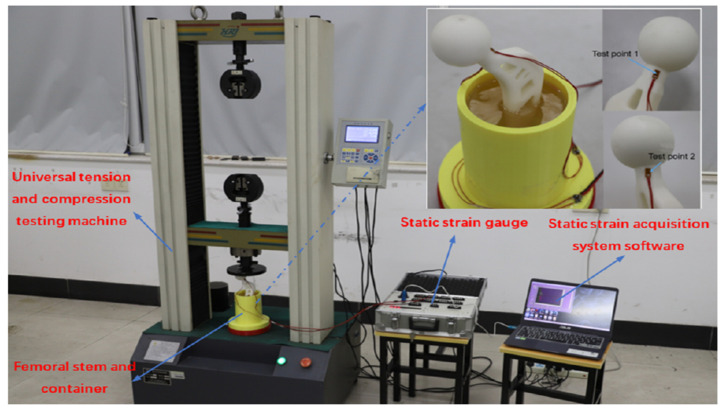
Test piece and experimental equipment.

In accordance with the ISO7206-4 standard, the fatigue performance of femur stem components is tested. The femur stem material used in the experiment is white resin with an elastic modulus of 2650 MPa and a Poisson’s ratio of 0.40. At the same time, the embedding medium of the femur stem used in this experiment is selected as filled epoxy resin, with an elastic modulus of 4000 MPa and a Poisson’s ratio of 0.39. The fixed container is processed and manufactured via 3D printing technology. Before packaging, the fixed container and prosthetic femur stem are cleaned. Through the fixture, clamping the femoral head into a fixed container handle and adjusting the former angle should be $10^{\circ }$; the deflection angle should be $9^{\circ }$. The test piece and experimental equipment are shown in Figure 11.

According to ISO7206-4, the CT value of the femoral shaft (the distance from the femoral head bulb to the farthest end of the femoral shaft) is between 120mm and 250mm and the distance from the upper surface to the femoral head bulb after the embedding medium is solidified should be 80mm. Since the connecting wire and the working time of the strain gauge are long in the loading test, the influence of the attachment strain value generated by the resistance strain gauge when the temperature changes on the strain data should be considered. In this regard, the wire temperature compensation formula is given by

(16) $$\varepsilon_{r1}=\frac{r_{e}}{R_{g}+r_{e}}\cdot \frac{\alpha }{K_{s}}$$

where $\varepsilon_{r1}$ is additional strain due to conductor temperature, $R_{g}$ is the resistance value of strain gauge, $r_{e}$ is the resistance value of wire, α is the resistance temperature coefficient of the wire and $K_{s}$ is the set strain rate of the strain measuring instrument. The strain values of the two test points are obtained by modifying the experimental data, as shown in Table 3.

### 5.2. Finite Element Analysis

**Figure 12:**
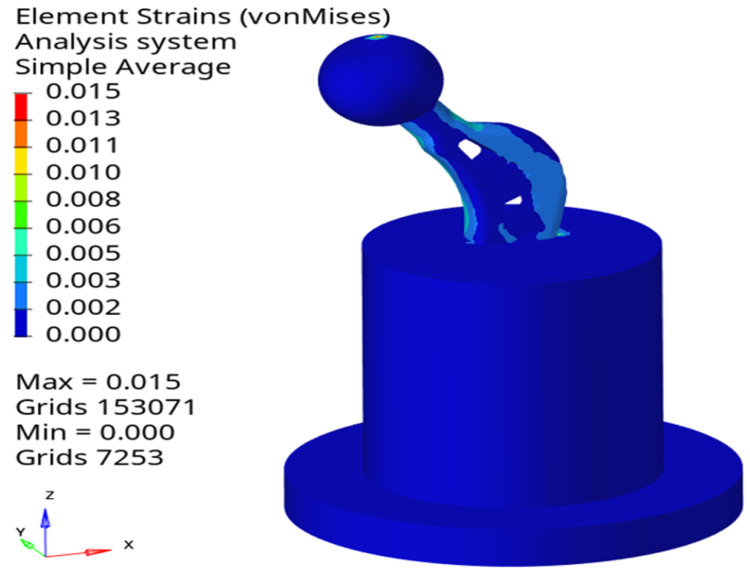
Overall stain nephogram of the proposed femoral stem.

Using Hypermesh software, the bottom of the packaging container is set as fixed, the contact between various components is set as rigid contact and a uniform vertically downward load with 400 N is applied to the head of the femoral stem. The overall strain nephogram is obtained, as shown in Figure 12.

**Figure 13:**
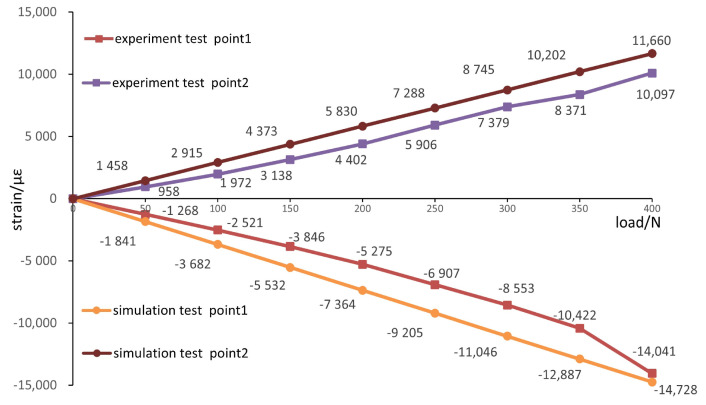
Comparison of experimental and simulated strain data.

Considering the simulation strain data could not be directly compared with the experimental data, the linear strain component of the simulation results should be calculated first and then the linear strain value at the test points should be determined. The strain value can be calculated by

(17) $$\varepsilon_{r}=\varepsilon_{x}l_{x}^{2}+\varepsilon_{x}l_{x}^{2}+\varepsilon_{x}l_{x}^{2}+\varepsilon_{xy}l_{x}l_{y}+\varepsilon_{yz}l_{y}l_{z}+\varepsilon_{zx}l_{z}l_{x}$$

where $l_{x}$, $l_{y}$ and $l_{z}$ are the cosine of the *x*, *y* and *z* directions, respectively. Finite element analysis is carried out for the loading conditions of 50 N, 100 N, 150 N, 200 N, 250 N, 300 N and 350 N successively and the linear strain values at the test points of numerical simulation are calculated through (17). A comparison with the experimental results is shown in Figure 13.

Figure 13 shows that measuring point 1 is the compressive strain and point 2 is the tensile strain. When the load is 300 N, the maximum error between the experimental strain value and the simulation value is −2493με and the relative error is 22.5%. The mean error is −1682με and the mean relative error is 20.3%. When the load is 350 N, the maximum error between the experimental strain value and the simulation value is 1831με and the relative error is 17.9%. The mean error is 1281με and the mean relative error is 19.5%. The strain values of the experimental results are all lower than those of the simulation. The main factors that caused the errors between the experimental results and the simulation values were as follows: the errors generated during measurement; bubbles were attached to the surface of the femoral stalk during the curing process of the packed epoxy resin. A gap between the fixed container and the universal tension and pressure testing machine platform influences the meshing accuracy in finite element analysis.

In the present work, only the influence of load direction change on structure stability is considered. In practical engineering design, it may be necessary to consider the structural stability changes caused by material loss and local fracture. Due to the differences in the daily living environment of different individuals, it may be necessary to consider the stress–strain analysis of the femoral stalk under conditions such as walking with gait and climbing stairs. We will do further research in this area in the future.

## 6. Conclusions

In this paper, a method combining the parallel topology optimization technique with the structural design of the prosthesis femur stem was proposed to obtain a new type of femur stem, namely, type B, and the SIMP topology optimization method was used to obtain type A femur stem. In addition, a multi-working condition stress analysis was carried out on both of them, providing a reference for the design of the porous femur stem. Key findings include the following:

(1) The stress and strain distributions of the femur corresponding to the prototype, type A and type B femoral stem structures under one-leg standing, abduction and adduction conditions were analyzed. The stress distribution of the femur corresponding to the B-type femoral stem was more similar to that of the healthy femur and the average value of the femur corresponding to the B-type femoral stem was the most similar to that of the healthy femur under the three conditions. The difference is 1.97 MPa, 3.11 MPa and 7.08 MPa, respectively.

(2) A B-type femur stem structure loading simulation experiment was carried out and compared with the simulation analysis results. The research results show that the average error between the experimental strain value and the simulation value is −1682με and the average relative error is 20.3% for the internal test points. For the measured points, the average error between the experimental and simulated strain values is 1281με and the average relative error is 19.5%.

## Figures and Tables

**Table 1:** Loads on the proximal femur under three operating conditions.

Condition	Joint Contact Force	Abductor Muscles
Stand	$24^{\circ }$ 2317 N	$28^{\circ }$ 703 N
Outreach	$-15^{\circ }$ 1158 N	$-8^{\circ }$ 351 N
Adduction	$56^{\circ }$ 1548 N	$35^{\circ }$ 468 N

**Table 2:** Average cross-section stress values under different conditions MPa.

	Section	One Leg Stand	Abduction	Adduction
Type A	A	20.68	15.78	8.92
	B	15.42	24.56	15.56
	C	8.32	27.31	26.34
Prototype	A	21.78	15.24	7.98
	B	18.53	25.61	17.36
	C	17.22	28.93	25.84
Type B	A	10.33	16.19	7.23
	B	12.54	22.13	16.57
	C	9.82	24.46	25.71
Healthy fermur	A	10.61	13.76	3.24
	B	6.21	20.52	16.23
	C	9.95	19.13	8.83

**Table 3:** Strain values of two test points under different loads.

	Load/N	Strain/με		Load/N	Strain/με
	50	−1268		50	958
	100	−2521		100	1972
	150	−3846		150	3138
$1^{\#}$	200	−5275	$2^{\#}$	200	4402
	250	−6907		250	5906
	300	−8553		300	7379
	350	−10,422		350	8371

## Data Availability

The corresponding author will provide the data used in this work upon reasonable request.
